# Supplementary material for: Human health risk assessment of edible body parts of chicken through heavy metals and trace elements quantitative analysis
Source: PLoS One. 2023 Mar 10;18(3):e0279043. doi: 10.1371/journal.pone.0279043 (PMC10004613; doi:10.1371/journal.pone.0279043)
Supplement: Attachment — Submitted filename: Response to Reviewers.docx [file pone.0279043.s002.docx]

Dear Dr. Mathilde Body-Malapel
Academic Editor, PLOS ONE

Thank you for allowing us to submit a revised draft of our manuscript titled “Quantitative analysis of heavy metals and trace elements in the edible body parts of the chicken and risk assessment to human health” to PLOS ONE. We appreciate the time and effort you and the reviewers have dedicated to providing valuable feedback on our manuscript. We are grateful to the reviewers for their insightful comments. We have incorporated changes to reflect most of the suggestions provided by the reviewers. We have highlighted the changes within the manuscript.

**Journal requirements:**

• **Comment 1**: *Please ensure that your manuscript meets PLOS ONE's style requirements, including those for file naming. The PLOS ONE style templates can be found at*

[*https://journals.plos.org/plosone/s/file?id=wjVg/PLOSOne_formatting_sample_main_body.pdf*](https://journals.plos.org/plosone/s/file?id=wjVg/PLOSOne_formatting_sample_main_body.pdf)  *and*

[*https://journals.plos.org/plosone/s/file?id=ba62/PLOSOne_formatting_sample_title_authors_affiliations.pdf*](https://journals.plos.org/plosone/s/file?id=ba62/PLOSOne_formatting_sample_title_authors_affiliations.pdf)

**Response:** Thank you for the observation. The revised manuscript has been prepared following PLOS ONE style templates.

• **Comment 2**: *Thank you for stating the following financial disclosure: “No manuscripts currently in press or under consideration elsewhere. The author(s) received no specific funding for this work. “At this time, please address the following queries:*

*a) Please clarify the sources of funding (financial or material support) for your study. List the grants or organizations that supported your study, including funding received from your institution.*

*b) State what role the funders took in the study. If the funders had no role in your study, please state: “The funders had no role in study design, data collection and analysis, decision to publish, or preparation of the manuscript.”*

*c) If any authors received a salary from any of your funders, please state which authors and which funders.*

*d) If you did not receive any funding for this study, please state: “The authors received no specific funding for this work.”*

*Please include your amended statements within your cover letter; we will change the online submission form on your behalf.*

**Response:** All amended statements are incorporated within the cover letter.

• **Comment 3**: *Thank you for stating the following in your Competing Interests section:*

*“NO authors have competing interests” Please complete your Competing Interests on the online submission form to state any Competing Interests. If you have no competing interests, please state "The authors have declared that no competing interests exist.", as detailed online in our guide for authors at* [*http://journals.plos.org/plosone/s/submit-now*](http://journals.plos.org/plosone/s/submit-now)

*This information should be included in your cover letter; we will change the online submission form on your behalf.*

**Response:** This information has been included in the cover letter.

• **Comment 4**: *Please update your submission to use the PLOS LaTeX template. The template and more information on our requirements for LaTeX submissions can be found at* [*http://journals.plos.org/plosone/s/latex*](http://journals.plos.org/plosone/s/latex)*.*

**Response:** We like to submit the LaTeX version of the manuscript after acceptance of the manuscript. We need external support to follow the PLOS LaTex template.

• **Comment 5**: *We note that Figure 1 in your submission contain [map/satellite] images which may be copyrighted. All PLOS content is published under the Creative Commons Attribution License (CC BY 4.0), which means that the manuscript, images, and Supporting Information files will be freely available online, and any third party is permitted to access, download, copy, distribute, and use these materials in any way, even commercially, with proper attribution. For these reasons, we cannot publish previously copyrighted maps or satellite images created using proprietary data, such as Google software (Google Maps, Street View, and Earth). For more information, see our copyright guidelines:* [*http://journals.plos.org/plosone/s/licenses-and-copyright*](http://journals.plos.org/plosone/s/licenses-and-copyright)*.*

*We require you to either (1) present written permission from the copyright holder to publish these figures specifically under the CC BY 4.0 license, or (2) remove the figures from your submission:*

*a. You may seek permission from the original copyright holder of Figure 1 to publish the content specifically under the CC BY 4.0 license.*

*We recommend that you contact the original copyright holder with the Content Permission Form (*[*http://journals.plos.org/plosone/s/file?id=7c09/content-permission-form.pdf*](http://journals.plos.org/plosone/s/file?id=7c09/content-permission-form.pdf)*) and the following text:*

*“I request permission for the open-access journal PLOS ONE to publish XXX under the Creative Commons Attribution License (CCAL) CC BY 4.0 (*[*http://creativecommons.org/licenses/by/4.0/*](http://creativecommons.org/licenses/by/4.0/)*). Please be aware that this license allows unrestricted use and distribution, even commercially, by third parties. Please reply and provide explicit written permission to publish XXX under a CC BY license and complete the attached form.”*

*Please upload the completed Content Permission Form or other proof of granted permissions as an "Other" file with your submission.*

*In the figure caption of the copyrighted figure, please include the following text: “Reprinted from [ref] under a CC BY license, with permission from [name of publisher], original copyright [original copyright year].”*

*b. If you are unable to obtain permission from the original copyright holder to publish these figures under the CC BY 4.0 license or if the copyright holder’s requirements are incompatible with the CC BY 4.0 license, please either i) remove the figure or ii) supply a replacement figure that complies with the CC BY 4.0 license. Please check copyright information on all replacement figures and update the figure caption with source information. If applicable, please specify in the figure caption text when a figure is similar but not identical to the original image and is therefore for illustrative purposes only.*

*The following resources for replacing copyrighted map figures may be helpful:*

*USGS National Map Viewer (public domain):* [*http://viewer.nationalmap.gov/viewer/*](http://viewer.nationalmap.gov/viewer/)

*The Gateway to Astronaut Photography of Earth (public domain):* [*http://eol.jsc.nasa.gov/sseop/clickmap/*](http://eol.jsc.nasa.gov/sseop/clickmap/)

*Maps at the CIA (public domain):* [*https://www.cia.gov/library/publications/the-world-factbook/index.html*](https://www.cia.gov/library/publications/the-world-factbook/index.html) *and* [*https://www.cia.gov/library/publications/cia-maps-publications/index.html*](https://www.cia.gov/library/publications/cia-maps-publications/index.html)

*NASA Earth Observatory (public domain):* [*http://earthobservatory.nasa.gov/*](http://earthobservatory.nasa.gov/)

*Landsat:* [*http://landsat.visibleearth.nasa.gov/*](http://landsat.visibleearth.nasa.gov/)

*USGS EROS (Earth Resources Observatory and Science (EROS) Center) (public domain):* [*http://eros.usgs.gov/#*](http://eros.usgs.gov/)

*Natural Earth (public domain):* [*http://www.naturalearthdata.com/*](http://www.naturalearthdata.com/)

**Response:** Fig 1 has been prepared using QGIS software. No Satellite image has been used to prepare the map. QGIS files of the figure has been uploaded as a "Supporting Information Compress" file with the online submission.

Here is a point-by-point response to the reviewers’ comments and concerns.

**Comments from Reviewer #1**

• **Comment 1**: *The title is too long*

**Response:** Thank you for highlighting this. We agree with this comment; therefore, we have changed the title to ‘**Human health risk assessment of edible body parts of chicken through heavy metals and trace elements quantitative analysis.**’ This addition can be found on page 01 and lines 1–3 in the ‘Revised Manuscript with Track Changes.docx’.

• **Comment 2**: *conclusion is not true according result of this study (this study demonstrated that consumers are chronically exposed to elemental contamination with cancer-causing and noncarcinogenic effects.) because (The calculated THQ and total target hazard quotient (TTHQ) values were measured less than one, suggesting that the consumption of chicken meat has no carcinogenic danger to its consumers) please re write this conclusion.*

**Response:** Thank you for pointing this out. We agree with this comment and have rewritten the conclusion section. This change can be found on pages 25-26, lines 455–472 in the ‘Revised Manuscript with Track Changes.docx’.

• **Comment 3**: *for improve the introduction section, please refer to the following related studies:* [*https://doi.org/10.1080/03067319.2020.1743835*](https://doi.org/10.1080/03067319.2020.1743835)*.* [*https://doi.org/10.1016/j.envres.2021.112002*](https://doi.org/10.1016/j.envres.2021.112002)*.*
[*https://doi.org/10.1080/10807039.2018.1460798*](https://doi.org/10.1080/10807039.2018.1460798)*.* [*https://doi.org/10.3390/ijerph16214261*](https://doi.org/10.3390/ijerph16214261)

**Response:** We agree with this comment and have incorporated suggested references in the introduction part. These changes can be found on page 3, lines 47-63, and page 10, lines 193 in the ‘Revised Manuscript with Track Changes.docx’.

• **Comment 4**: *demonstrate novelty in the end paragraph part of introduction.*

**Response:** Thank you for pointing this out. We agree with this comment and have rewritten the end paragraph part of the introduction. This change can be found on page 05, lines 87-95 in the ‘Revised Manuscript with Track Changes.docx’.

• **Comment 5**: *Where are these analyzes used and where are their results reported? A one-way analysis of variance (ANOVA) test was used to get levels of heavy metal variations in the tested chicken edible body parts that were statistically significant. The t-test was used to determine the statistically significant differences between the heavy metals. What’s the sampling strategy?*

**Response:** We agree with this comment and have deleted two confusing lines from the manuscript. A multistage cluster sampling strategy was used in the research.

• **Comment 6**: *use this reference in method section: Health risk assessment of heavy metals in cosmetic products sold in Iran: the Monte Carlo simulation. Health risk assessment due to fluoride exposure from groundwater in rural areas of Agra, India: Monte Carlo simulation.*

**Response:** We agree with this comment and have incorporated the reference in the method section. This change can be found on page 11, line 224 in the ‘Revised Manuscript with Track Changes.docx’.

• **Comment 7**: *The conclusion should be rewritten. It is essential to rewrite the conclusion to summary primary results, primary viewpoint, shortcomings, academic contribution, and some suggestion.*

**Response:** Thank you for pointing this out. We agree with this comment and have rewritten the conclusion section. This change can be found on pages 25-26, lines 455–472 in the ‘Revised Manuscript with Track Changes.docx’.

**Comments from Reviewer #2**

• **Comment 1**: *Some non-technical words such as dangerous heavy metals may be replaced by toxic heavy metals, cancer-causing by carcinogenic, and so on.*

**Response:** Thank you for highlighting this. We agree with this comment; therefore, the word ‘dangerous heavy metals’ have been replaced by toxic heavy metals, and ‘cancer-causing’ has been replaced by carcinogenic. These changes have been made throughout the manuscript.

• **Comment 2**: *Heavy metals have a specific weight greater than 5 g/cm3 and are naturally present in the Earth’s crust. Ref. is missing here insert (*[*https://doi.org/10.1080/02772248.2017.1413652*](https://doi.org/10.1080/02772248.2017.1413652)*)*

**Response:** We agree with this. Accordingly, we have included the reference. This change can be found on page 03, line 47 in the ‘Revised Manuscript with Track Changes.docx’.

• **Comment 3**: *Ref. 3 for the sentence “Heavy metals linger in the environment for a long time because they are difficult to break down” is not appropriate, the correct reference could be* [*https://doi.org/10.1155/2019/6730305*](https://doi.org/10.1155/2019/6730305)

**Response:** Thank you for pointing this out. We agree with this comment; therefore, the correct reference is used in the revised manuscript. This change can be found on page number 03 and lines 53-54 in the ‘Revised Manuscript with Track Changes.docx’.

• **Comment 4**: *Check symbols of the metals being studied, throughout the text. Wrote their full name if you want, at their first mention, in the following text only symbols are enough.*

**Response:** We agree. Thank you for highlighting this. Accordingly, the symbols of the metals have been checked and rewritten in the revised manuscript.

• **Comment 5**: *Citation style in the text is not appropriate, please check throughout the entire text. For instance, “e.g., [2], [7], [9], [14], [15], [19], [26], [27], [29], [31], [32], [37]” references for one statement are too much. Although it is less than [59] Iwegbue et al. (2008) (0.013.22 mg.kg-1), the Pb concentration in chicken gizzards from the Rayer Bazar region was greater than those in chicken gizzards from any other location, check and rephrase; with the study of [37]; according to [38]; in [39]; in [36]. “obtained by [59] Iwegbue et al., (2008)(6.12-33.21 mg/kg), [55] Alturiqi & Albedair, (2012)(27.93-36.93 mg/kg), and [61] Rahman et al., (2014) (26.67±0.25-28.67±0.34 mg/kg).”*

**Response:** Thank you for highlighting this. We agree with this comment; therefore, the citation style in the text has also been changed in the revised manuscript. This change can be found on page number 06, line 126 in the ‘Revised Manuscript with Track Changes.docx’.

• **Comment 6**: *At various places the word “percent” must be replaced by %, check all other units according standard international protocol. per Sq. Km. is better to be replaced by km-2.*

**Response:** We agree with this and have incorporated your suggestion; “percent” has been replaced by % and per Sq. Km. has been replaced by km-2.

• **Comment 7:** *“Whatman filter paper (number 42) after the samples” check specification of the filter paper and represent in line with other reports.*

**Response:** Thank you so much for your comment. We have checked, and the number is 42.

• **Comment 8:** *Table 1, contains some columns which is mere repetition of the identical statements, omit these columns, column 5 and 7 needs to be deleted.*

**Response:** Thank you for highlighting this. We agree with this comment. Accordingly, we have fixed the table. This change can be found on page 8 and line 164 in the ‘Revised Manuscript with Track Changes.docx’.

• **Comment 9:** *Table 2 is very short better to merge it in the text.*

**Response:** We agree with you. Thank you for highlighting this. Therefore, we have merged it in the text. This change can be found on page 10 and lines 199-200 in the ‘Revised Manuscript with Track Changes.docx’.

• **Comment 10:** *Some sentences are very short, such as “Lead is known to be a carcinogen”*

**Response:** We agree with this and have rewritten the sentences. This change can be found on page 15 and line 259 in the ‘Revised Manuscript with Track Changes.docx’.

• **Comment 11:** *Mostly the table is empty, what is need of such tables? Remove or modify only for valued data, It”s removal is highly recommended*

**Response:** Thank you for highlighting this. We modified the table for valued data.

• **Comment 12:** *Reference position of Table 5 is appropriate not in Table 4.*

**Response:** We agree with this and have corrected it accordingly. This change can be found on page 18 and line 332 in the ‘Revised Manuscript with Track Changes.docx’.

• **Comment 13:** *Punctuations at several places are inappropriate such as “results of [47]. [14], but” and several other places.*

**Response:** Thanks for the observation. We agree with this and have checked and corrected the punctuation throughout the manuscript.

• **Comment 14:** *Some numerical data is needed to be included in the conclusion.*

**Response:** We agree with this and have incorporated some numerical data in the conclusion section. This change can be found on pages 25-26, lines 455–472 in the ‘Revised Manuscript with Track Changes.docx’.

• **Comment 15:** 15. Check references for uniform format and remove some irrelevant references like Ref. [3] does not fit to the statement.

**Response:** We agree with the observation. A relevant reference has replaced the reference [3]. This change can be found on page 03 and lines 53–54 in the ‘Revised Manuscript with Track Changes.docx’.

**Additional clarifications**

In addition to the above comments, all spelling and grammatical errors highlighted by the reviewers have been corrected.

We look forward to hearing from you regarding our submission and responding to any further questions and comments you may have.

Sincerely,

Syed Hafizur Rahman

Date: 23-11-2022
